# Activation of A2A Receptor by PDRN Reduces Neuronal Damage and Stimulates WNT/β-CATENIN Driven Neurogenesis in Spinal Cord Injury

**DOI:** 10.3389/fphar.2018.00506

**Published:** 2018-05-29

**Authors:** Natasha Irrera, Vincenzo Arcoraci, Federica Mannino, Giovanna Vermiglio, Giovanni Pallio, Letteria Minutoli, Gianluca Bagnato, Giuseppe Pio Anastasi, Emanuela Mazzon, Placido Bramanti, Francesco Squadrito, Domenica Altavilla, Alessandra Bitto

**Affiliations:** ^1^Department of Clinical and Experimental Medicine, AOU Policlinico G. Martino, University of Messina, Messina, Italy; ^2^Department of Biomedical and Dental Sciences and Morphological and Functional Sciences, AOU Policlinico G. Martino, University of Messina, Messina, Italy; ^3^NIHR Leeds Biomedical Research Centre, Leeds Teaching Hospitals NHS Trust and Leeds Institute of Rheumatic and Musculoskeletal Medicine, University of Leeds, Leeds, United Kingdom; ^4^IRCCS Centro Neurolesi “Bonino-Pulejo”, Messina, Italy

**Keywords:** adenosine receptors, spinal cord injury, polydeoxyribonucleotide, inflammation, neurogenesis

## Abstract

Spinal cord injury (SCI) is a complex clinical and progressive condition characterized by neuronal loss, axonal destruction and demyelination. In the last few years, adenosine receptors have been studied as a target for many diseases, including neurodegenerative conditions. The aim of this study was to investigate the effects of an adenosine receptor agonist, PDRN, in an experimental model of SCI. Moreover, since adenosine receptors stimulation may also activate the Wnt pathway, we wanted to study PDRN effects on Wnt signaling following SCI. Spinal trauma was induced by extradural compression of spinal cord at T5-T8 level in C57BL6/J mice. Animals were randomly assigned to the following groups: Sham (*n* = 10), SCI (*n* = 14), SCI+PDRN (8 mg/kg/i.p.; *n* = 14), SCI+PDRN+DMPX (8 and 10 mg/kg/i.p., respectively; *n* = 14). DMPX was used as an adenosine receptor antagonist to evaluate whether adenosine receptor block might prevent PDRN effects. PDRN systemically administered 1 h following SCI, protected from tissue damage, demyelination, and reduced motor deficits evaluated after 10 days. PDRN also reduced the release of the pro-inflammatory cytokines TNF-α and IL-1β, reduced BAX expression and preserved Bcl-2. Furthermore, PDRN stimulated Wnt/β-catenin pathway and decreased apoptotic process 24 h following SCI, whereas DMPX administration prevented PDRN effects on Wnt/β-catenin signaling. These results confirm PDRN anti-inflammatory activity and demonstrate that a crosstalk between Wnt/β-catenin signaling is possible by adenosine receptors activation. Moreover, these data let us hypothesize that PDRN might promote neural repair through axonal regeneration and/or neurogenesis.

## Introduction

Spinal cord injury (SCI) is a complex clinical and progressive condition characterized by neuronal loss, axonal destruction and demyelination, in particular at the site of impact (Maegele et al., 2005). Injuries on spinal cord affect neurons which suffer from alterations such as atrophy and shrinkage (Brock et al., 2010; Nielson et al., 2011). Spinal cord atrophy is related to the decrease of cortico-spinal tract integrity and cortical gray matter volume, suggesting that SCIs activate degenerative processes that spread toward the brain (Freund et al., 2013).

When traumatic and mechanical injuries hit spinal cord, neurons start to die and tissue loses neuronal cells which cannot be recovered or regenerated. SCI is characterized by acute as well as progressive damage, which may worsen the prognosis of the disease (Hagg and Oudega, 2006). The traumatic injury causes death of cells nearby the site of impact and stretching of myelinated axons may cause membrane damage up to axon degeneration (Shi and Pryor, 2002). The above described processes start during the acute phase, continue during the chronic stage, eventually causing the atrophy of the spinal cord.

A “secondary” phase generally follows the mechanical injury characterized by microvascular dysfunction and enrolment of inflammatory cells. Once activated, inflammatory cells release pro-inflammatory cytokines, which contribute to amplify spinal cord damage. Local and systemic inflammation is responsible for neurodegenerative processes during both acute and chronic phases of SCI, and consequently leads to death of glial cells and neurons, leaving glial scar and a cavity in the spinal parenchyma (Fleming et al., 2006; Kigerl et al., 2009). Indeed, leukocyte infiltration and production of cytokines such as tumor necrosis factor alpha (TNF-α) are markedly increased following SCI (Zhou et al., 2014). In particular, microglia/macrophages activation associated with inflammatory process seems to be an important factor of neuronal degeneration and regeneration (Beuche and Friede, 1984; Zhou et al., 2014). Furthermore, recent evidences suggest that SCI may cause wingless-type mouse mammary tumor virus integration site family (Wnt) signaling down-regulation and, as a matter of fact, one of its inhibitors Wif1 is highly expressed following SCI, in particular from hours to days post-injury (Fernández-Martos et al., 2011).

The Wnt/β-catenin pathway regulates several processes during normal organ development and tissue homeostasis (Clevers, 2006) and alterations of this pathway are involved in various diseases (Cheon et al., 2006; Beyer et al., 2012; Guo et al., 2012), including neurodegenerative ones.

Currently, drugs prescribed to treat SCI, such as corticosteroids, are used to prevent secondary inflammatory neuronal damage (Moreno-Flores and Avila, 2006) and to improve behavioral outcomes (Hurlbert, 2006). These medical strategies are not 100% effective in treating SCI, but are useful to ameliorate symptoms; for this reason, the researchers need to focus their attention on others molecules and targets to manage patients suffering consequences of SCI. In this context, adenosine A2A receptor activation by ATL-146e, a selective adenosine A2A agonist, has been proved effective in reducing both neutrophil recruitment and TNF-α release, improving locomotor dysfunction and neurological function in a rabbit SCI-model (Cassada et al., 2002). Furthermore, it has been demonstrated that adenosine A2A receptor stimulation protects spinal cord by its vasodilatory effects on neural microvasculature (Vinten-Johansen et al., 2003). In particular, the A2A receptor plays a central role in halting inflammation, in fact TNF-α and other pro-inflammatory cytokines increase A2A receptor function by preventing receptor desensitization, further downregulating inflammation (Khoa et al., 2006). In the last few years, adenosine receptors have been studied as a target for many diseases, including neurodegenerative conditions (Burnstock, 2016); considering these previous observations, adenosine receptor agonists might represent a promising group of candidate compounds for the management of SCI. Polydeoxyribonucleotide (PDRN) is a natural occurring A2A agonist that has been proven effective in reducing inflammation and improving tissue regeneration, thus its protective effects were investigated in an experimental model of SCI.

## Materials and Methods

### Animals

Adult female C57BL6/J mice (25–30 g; Charles River, Calco, Italy) were used in this study. Animals were housed in plastic cages, maintained under controlled environmental conditions (12 h light–dark cycle, 24°C), and provided with standard food and water *ad libitum* in the Animal Facility of the Department of Clinical and Experimental Medicine of the University of Messina.

All experiments were carried out according to the standards for care and use of animals as stated in the Directive 2010/63/EU, and the ARRIVE guidelines (Kilkenny et al., 2010).

The procedures were evaluated and approved by the Ethic Committee of the University of Messina (OPBA, Approval No. #09/14).

Animals were randomly assigned to the following groups: Sham (*n*= 10), SCI (*n* = 14), SCI + PDRN (8 mg/kg/i.p.; *n* = 14), SCI+PDRN+DMPX (10 mg/kg/i.p.; *n* = 14). DMPX (3,7-Dimethyl-1-propargylxanthine) is an A2A receptor antagonist and was administered with PDRN to demonstrate PDRN mechanism of action on A2A receptor. PDRN and DMPX were administered 1 h following SCI.

Animals from each group (five Sham and seven mice from all other groups) were randomly selected for being killed at either 24 h or 10 days after surgery.

### Experimental Spinal Cord Injury

Mice were anesthetized with ketamine/xylazine at the dose of 80 and 10 mg/kg/i.p., respectively. The experimental model of SCI was firstly described by Rivlin and Tator in the “clip compression model” (Rivlin and Tator, 1978). SCI was induced by extradural compression of a section of the spinal cord exposed through a four-level T5-T8 laminectomy and the prominent spinal process of T-5 was used as a surgical guide. An aneurysm clip was extradurally applied at T5-T8 level for 60 s with a closing force of 24 g. Sham-injured mice were subjected to laminectomy but aneurysm clip was not applied. Following Sham or SCI procedures the muscular and skin layers were sutured with 4-0 silk thread and saline solution was administered to replace the blood volume.

### Locomotor Performance

Locomotor performance was analyzed using the Basso mouse scale (BMS) open-field score (Basso et al., 1995). The test was performed to assess the degree of motor dysfunction following SCI. All mice were allowed to walk in an open field box before SCI induction, to accustom animals to the new space. Mice were tested before injury and each day following SCI (for 4 min by two blinded observers), for up to 10 days.

Scores were assigned on a scale of 0–9: (0) completely paralyzed; (1–2) slight (<90°) to widespread (>90°) ankle movement; (3–4) ranges from sporadic to frequent dorsal stepping to occasional plantar stepping; (5) frequent to consistent plantar stepping and loss of coordination with rotated paw placement; (6–7) frequent to consistent stepping with some to mostly coordinated stepping with paw rotation and severe trunk instability; (8) almost normal stepping with points taken off for trunk instability and whether the tail was up or down; (9) normal coordination, paw placement and trunk stability.

### Quantification of Circulating IL-1β and TNF-α

Blood was collected 24 h following SCI to evaluate IL-1β and TNF-α levels in serum, using specific ELISA kits (Cusabio, College Park, MD, United States). Samples were run in duplicate, and the absorbance was read at 450 nm; the results were compared to the standard curves and expressed as pg/mL.

### Measurement of Myeloperoxidase Activity

Myeloperoxidase (MPO), a marker of polymorphonuclear leukocyte accumulation, was determined as previously described (Mullane et al., 1985). Equal amounts of spinal cord tissue were homogenized mechanically with the MICCRA D-1 homogenizer (Miccra Gmbh, Müllheim, Germany), in a solution containing 0.5% hexa-decyl-trimethylammonium bromide dissolved in 10 mM potassium phosphate buffer (pH 7.0). Lysates were then centrifuged for 30 min at 15000 rpm at 4°C. Supernatant was incubated with a solution of 1.6 mM tetra-methyl-benzidine and 0.1 mM H_2_O_2_. The absorbance was measured at 650 nm. MPO activity was defined as the quantity of enzyme degrading 1 μmol hydrogen peroxide/min at 37° and was expressed as units per gram of tissue.

### Histology

Spines were removed from animals and fixed in 10% buffered formalin for 12 h, then vertebras were removed and the tissue was embedded in paraffin, and cut at 5 μm. Slides were rehydrated in graded ethanol, stained with hematoxylin and eosin, re-dehydrated in ascending graded ethanol, mounted with coverslips and observed with a light microscope at different magnifications.

Damaged neurons were counted also evaluating gray matter alterations. A six-point scale was used (Sirin et al., 2002): (0) no lesion observed; (1) gray matter contained 1 to 5 eosinophilic neurons; (2) gray matter contained 5 to 10 eosinophilic neurons; (3) gray matter contained more than 10 eosinophilic neurons; (4) small infarction with less than one third of the gray matter area; (5) moderate infarction if one third to one half of the gray matter area was involved; and (6) large infarction with the involvement of more than half of the gray matter area. An average of the scores for each group was recorded to obtain a cumulative score.

### Immunofluorescence

Consecutive sections of spinal cord tissues, collected 24 h following SCI and prepared for histological analysis were rehydrated, immersed in sodium citrate buffer (10 mM Sodium citrate, 0.05% Tween 20, pH 6.0) and put at 750W in microwave 3 min for three times to retrieve the antigen. Slides were then immersed in phosphate buffered saline (PBS) and incubated with 1% bovine serum albumin (BSA), 0.3% Triton X-100 in PBS, for 20 min, at room temperature. Sections were then incubated with mouse monoclonal anti SMI-32 RT antibody (1:1000 dilution; Covance, Eteryville, CA, United States) for 1 h.

Primary antibody was detected using Texas Red-conjugated IgG anti mouse (1:100 dilution; Jackson ImmunoResearch Laboratories, West Grove, PA, United States) for 1 h in the dark at room temperature. Sections were incubated with DAPI (1:1000 dilution; Sigma Chemicals, St. Louis, MO, United States) for 10 min in the dark at room temperature to label nuclei and sealed with mounting medium.

Samples were observed with a Zeiss LSM 510 confocal microscope equipped with Argon laser (458 and 488 nm λ) and two HeNe laser (543 and 633 nm λ). All images were digitized at a resolution of eight bits into an array of 2048 × 2048 pixels. Optical sections of fluorescence specimens were obtained at 488 nm λ, at 62/s scanning speed with up to eight repetitions on average; the pinhole size was set for optimal resolution. Contrast and brightness were established by examining the most brightly labeled pixels and choosing settings that allowed clear visualization of structural details while keeping the highest pixel intensities near 200.

### qPCR Assays

Total RNA was extracted from spinal cord tissue 24 h following SCI using Trizol LS reagent (Invitrogen, Carlsbad, CA, United States), quantified with a spectrophotometer (NanoDrop Lite, Thermo Fisher) and 2.5 μg was reverse transcribed using the Superscript VILO kit (Invitrogen) in a volume of 20 μl. To evaluate gene expression 1 μl of cDNA was added to the EvaGreen qPCR Master Mix (Biotium, Inc., Fremont, CA, United States), in a volume of 20 μl per well and BAX (Fw: 5^′^-CGAGCTGATCAGAACCATCA-3^′^ Rv: 5^′^-CTCAGCCCATCTTCTTCCAG-3^′^), BCL2 (Fw: 5^′^-ATACCTGGGCCACAAGTGAG-3^′^ Rv: 5^′^-TGATTTGACCATTTGCCTGA-3^′^), Wnt3a (Fw: 5^′^-TTCTTACTTGAGGGCGGAGA-3^′^ Rv: 5^′^-CTGTCGGGTCAAGAGAGGAG-3^′^), DKK-1 (Fw: 5^′^-AATCGAGGAAGGCATCATTG-3^′^ Rv: 5^′^-GCTTGGTGCATACCTGACCT-3^′^) and β-catenin (Fw: 5^′^-CGGCACCTTCCTATTTCTTCT-3^′^ Rv:5^′^-TCTGGAAATTAACTTCAGGCAAAC-3^′^) were assayed. Samples were run in duplicate and GADPH (Fw: 5^′^-GTCAAGGCTGAGAATGGGAA-3^′^ Rv: 5^′^-ATACTCAGCACCAGCATCAC-3^′^) was used as housekeeping gene; the reaction was performed using the two-step thermal protocol as recommended by the manufacturer.

Results were calculated using the 2^-ΔΔC_t_^ method, and expressed as n-fold increase in gene expression using the CTRL group as calibrator.

### Statistical Analysis

All data are expressed as means ± SD. Comparisons between experimental groups were analyzed by one-way ANOVA for non-parametric variables with Tukey’s post-test for intergroup comparisons. The possibility of error was set at *p* < 0.05 and was considered as statistically significant. Graphs were drawn using GraphPad Prism (version 5.0 for Windows).

## Results

### PDRN Promoted Recovery of Motor Function

**FIGURE 1:**
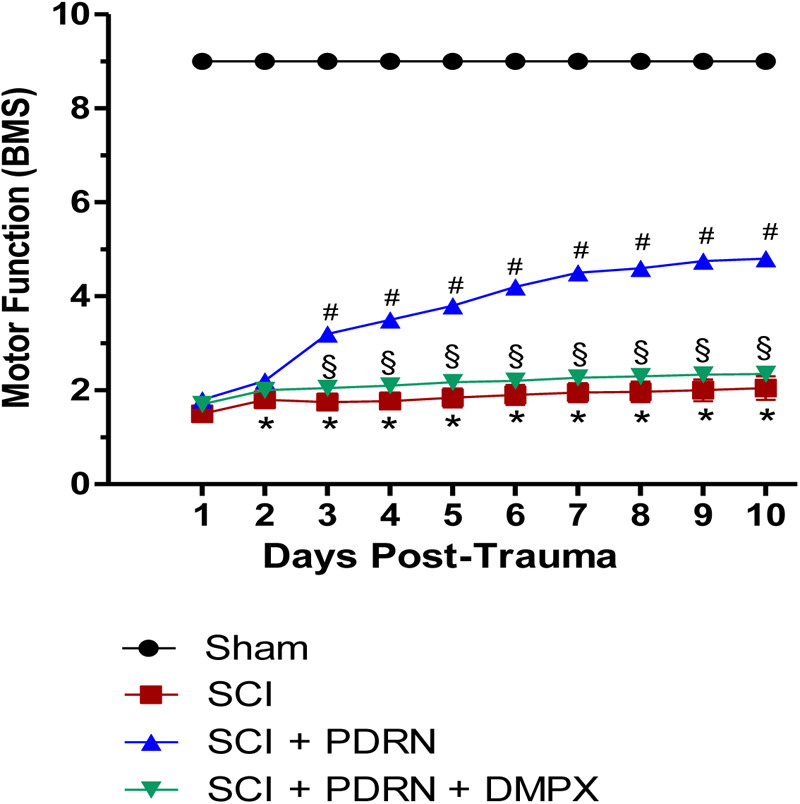
The graph represents the score of motor function of mice subjected to trauma. Recovery from motor dysfunction was graded using the Basso mouse scale (BMS). Data are expressed as means ± SD. Sham group included five mice, while all other groups included seven mice. ^∗^*p* < 0.05 vs. Sham; ^#^*p* < 0.05 vs. SCI; ^x^*p* < 0.05 vs. SCI+PDRN.

Motor function evaluated using the BMS demonstrated a significantly reduced locomotor activity in all SCI animals, compared to Sham animals, because of the loss of hindlimb movement. Sham-injured mice did not show motor impairment since they were only subjected to laminectomy and the aneurysm clip was not applied. PDRN treatment administered every 24 h for 10 days resulted in a significant improvement of motor function. Instead, DMPX administration abrogated motor recovery on mice treated with both PDRN and DMPX, blocking PDRN positive effects (**Figure 1**).

### Anti-inflammatory Effects of PDRN on Cytokine Release

**FIGURE 2:**
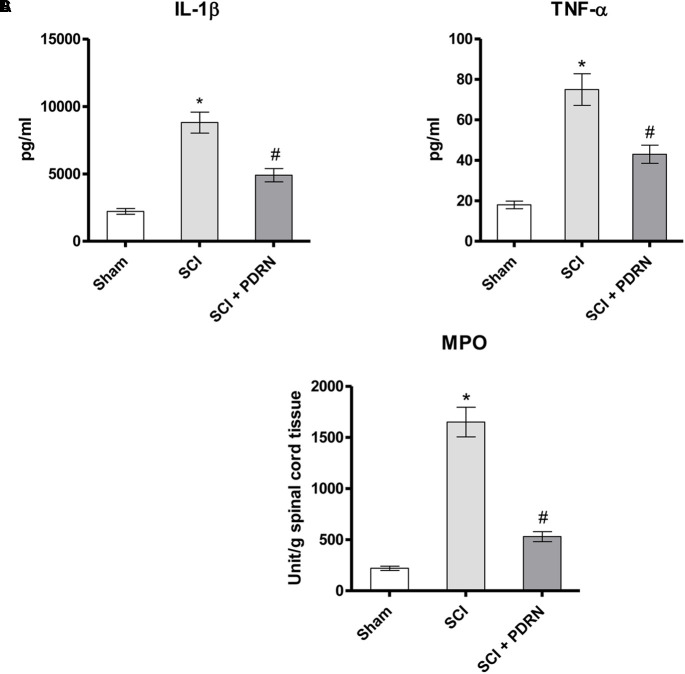
The graphs represent the levels of IL-1β **(A)** and TNF-α **(B)** in serum collected from each group 24 h after SCI. Levels of both pro-inflammatory cytokines were determined by enzyme-linked immunosorbent assay (ELISA). The graph **(C)** represents PDRN effects on myeloperoxidase activity from each group of animals 24 h following SCI. Values shown are expressed as the means and SD. Sham group included five mice, while all other groups included seven mice.^∗^*p* < 0.05 vs. Sham; ^#^*p* < 0.05 vs. SCI.

The pro-inflammatory IL-1β and TNF-α were markedly increased in serum of SCI animals 24 h following SCI as compared to Sham mice, which showed almost undetectable levels of these cytokines. PDRN administration markedly decreased both IL-1β and TNF-α levels in SCI-treated animals (**Figures 2A,B**; *F* = 229.3 and 179.3, factors = 3, respectively; *p* < 0.05), thus confirming PDRN anti-inflammatory property.

### Effects of PDRN on Neutrophil Infiltration

The severity of spinal cord inflammation is related to leukocytes infiltration into the spinal cord. Therefore, PDRN effect was also evaluated on neutrophil infiltration by measuring tissue MPO activity. MPO activity was increased in SCI group, as compared to Sham. PDRN administration significantly reduced polymorphonuclear granulocytes heap (**Figure 2C**; *F* = 450.7, factors = 3; *p* < 0.05), confirming the protective effects of PDRN on MPO activity also during SCI.

### PDRN Reduced the Severity of Secondary Spinal Cord Damage

**FIGURE 3:**
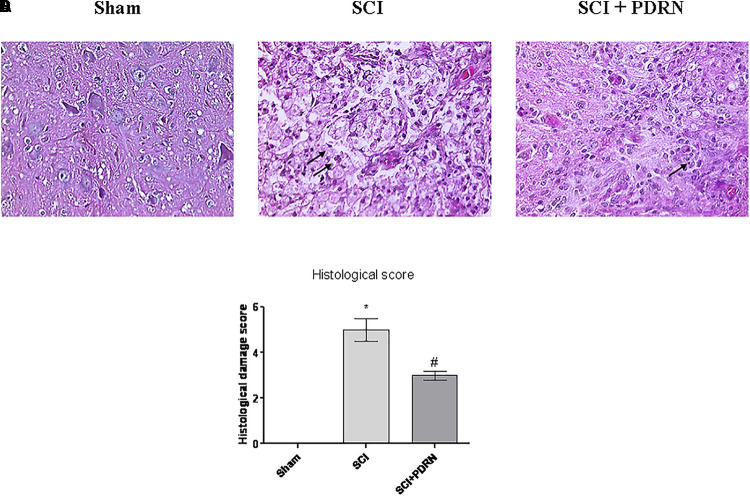
Representative H&E staining of spinal cord tissue from mice 24 h following SCI: **(A)** Sham, **(B)** SCI, and **(C)** SCI+PDRN. The arrows point at granulocyte infiltration. The graph in **(D)** represents the histological score. Data are expressed as means and SD. Sham group included five mice, while all other groups included seven mice. ^∗^*p* < 0.05 vs. Sham; ^#^*p* < 0.05 vs. SCI.

Spinal cord injury group showed a widespread damage both in the site of injury and in the perilesional area, as confirmed by the presence of oedema and inflammatory features, compared to Sham mice (**Figures 3A,B**). Histological results in terms of inflammation confirm the results of MPO activity, which indicated granulocyte infiltration. In fact, widespread granulocyte infiltration is extended to all the microscopic field, as demonstrated by the violet staining of the nuclei (representative arrows, **Figure 3B**). Moreover, spinal cord of SCI animals showed signs of neuronal suffering (vacuolization) with pycnotic areas and fragmented axons.

Granulocyte infiltration was decreased and contained in small areas (**Figure 3C**) following treatment with PDRN. Moreover, PDRN administration reduced oedema and preserved neurons from cell death (**Figure 3C**), suggesting that the treatment might be protective decreasing both inflammatory and cell death processes, at least 24 h following injury (**Figure 3D**; *F* = 458.6, factors = 3; *p* < 0.05).

### PDRN Preserved Neuron Structure

**FIGURE 4:**
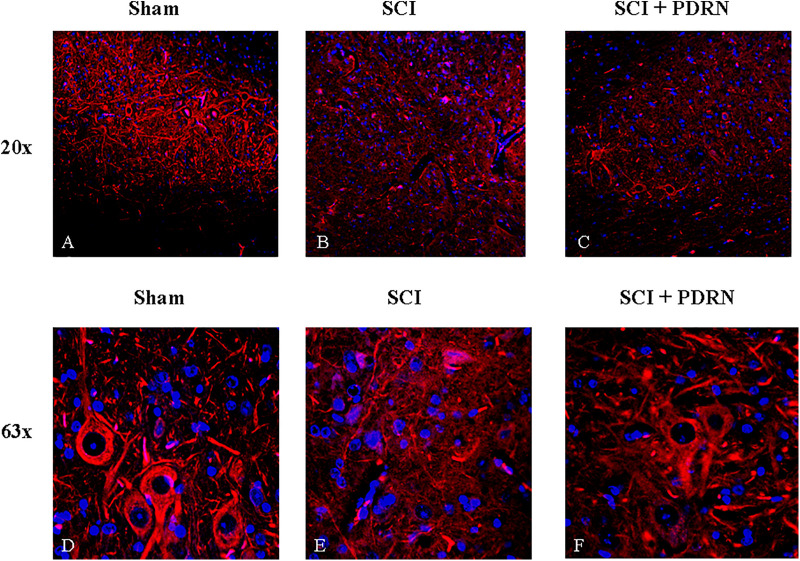
Representative images of spinal cord tissues showing immunofluorescence staining for SMI-32. **(A)** Spinal cord of sham animal. Original magnification 20X. **(B)** Spinal cord of SCI animal. Original magnification 20X. **(C)** Spinal cord of SCI animal treated with PDRN. Original magnification 20X. **(D)** Spinal cord of sham animal. Original magnification 63X. **(E)** Spinal cord of SCI animal. Original magnification 63X. **(F)** Spinal cord of SCI animal treated with PDRN. Original magnification 63X.

Sham animals showed a normal microscopic structure of the spinal cord with several neurons visibly connected to each other by long axonal and dendritic projection; neurons displayed a well-defined soma, long and thin axons as evidenced by SMI (Anti-Neurofilament H Mouse) antibody (red channel; **Figures 4A,D**) staining.

SMI staining pattern from SCI-group showed fragmented axonal and dendritic projections in the perilesional area of the spinal cord. Several fragments of interrupted axonal and dendritic projections were observed at high magnification and no neuronal soma in the perilesional area was detectable (**Figures 4B,E**). In addition, acute axonal degeneration was visible in the picture.

Spinal cord injury group treated with PDRN showed few neuronal somas positive for SMI in the perilesional area of spinal cord but fragments of interrupted axonal and dendritic projections were still detectable. PDRN conserved neuron structure, which showed a well-preserved soma with axons shortly interrupted from their origin. No dendritic projections were detectable (**Figures 4C,F**).

### PDRN Prevented Neuronal Death

**FIGURE 5:**
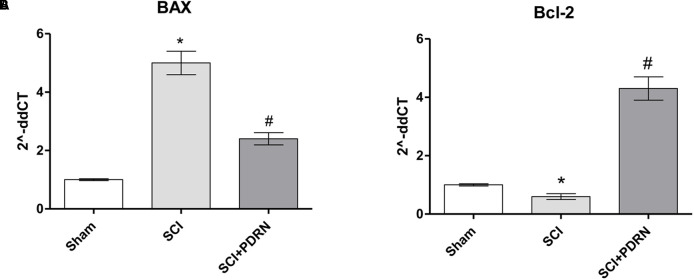
The graphs represent qPCR results of **(A)** BAX and **(B)** Bcl-2 from spinal cord tissues 24 h following SCI. ^∗^*p* < 0.05 vs. Sham; ^#^*p* < 0.05 vs. SCI. Data are expressed as means and SD.

At 24 h SCI animals showed increased apoptotic rate compared to Sham mice, as demonstrated by the markedly increased expression of the pro-apoptotic BAX protein, and a significant decrease of the Bcl-2 anti-apoptotic protein (**Figures 5A,B**). PDRN administration preserved neurons from apoptosis, in fact BAX expression was reduced, whereas Bcl-2 expression was increased at 24h, compared to SCI (**Figures 5A,B**; *F* = 421.7 and 506.0, factors = 3, respectively; *p* < 0.05).

### PDRN Modulated Wnt Signaling

**FIGURE 6:**
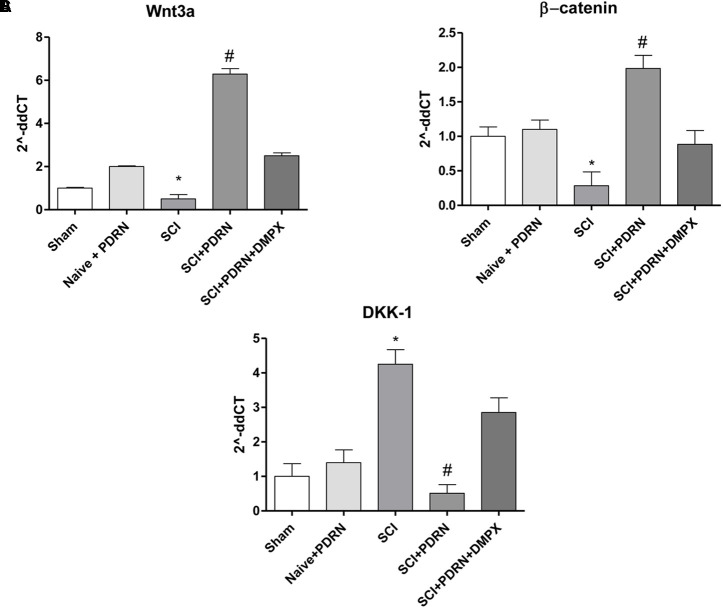
The graphs represent qPCR results of **(A)** Wnt3a, **(B)** β-catenin, and **(C)** DKK-1 from spinal cord tissues 24 h following SCI. Data are expressed as means and SD. Sham group included five mice, while all other groups included seven mice. ^∗^*p* < 0.05 vs. Sham; ^#^*p* < 0.05 vs. SCI.

Wnt3a mRNA expression and consequently β-catenin mRNA expression was dropped in SCI animals compared to Sham group, whereas treatment with PDRN significantly increased mRNA expression of both Wnt3a and β-catenin (**Figures 6A,B**).

In contrast, DKK-1, which is considered one of the most important Wnt inhibitors, demonstrated an increased mRNA expression in SCI animals compared to Sham mice. PDRN reduced DKK-1 mRNA expression in spinal cords of treated animals, confirming its effect on Wnt pathway activation (**Figure 6C**). DMPX abolished PDRN effects on Wnt signaling targets, thus demonstrating that PDRN mode of action was related to A2A receptor binding (**Figures 6A–C**; *F* = 2363, 157.2 and 221.9, factors = 5, respectively; *p* < 0.05).

## Discussion

Adenosine receptors are located in astrocytes, microglial cells, myelinated fibers, and neurons and their expression increases in injured spinal cord (Del Puerto et al., 2013). In addition, circulating cytokines as TNF-α might also increase the expression of adenosine receptors in a compensatory feedback manner (Kucher and Neary, 2005).

It is then rational to hypothesize the use of adenosine receptor agonists, to protect neurons following SCI. In addition, these agonists may also have advantages in clinical practice compared to corticosteroids in terms of therapeutic window and side effects. The mechanism of action of methylprednisolone is based on its anti-inflammatory and antioxidant properties by activation of transcriptional processes that require too long time. Instead, A2A receptor activation increases cAMP levels and exerts anti-inflammatory effects by seconds (Linden, 2001), inactivating neutrophils and reducing pro-inflammatory cytokines (Sullivan et al., 1990). In agreement with the fast-acting effect obtained with A2A receptor activation, PDRN systemically administered 1 h following SCI, protected from tissue damage, demyelination, and release of the pro-inflammatory cytokines TNF-α and IL-1β.

One of the first consequences following SCI is functional impairment characterized by loss of motor and/or sensory function in the upper and lower limbs and often also in the trunk (Kirshblum et al., 2011). A2A agonists administration may be effective in protecting from motor deficits; in fact, mice lacking A2A receptors on bone marrow-derived cells (BMDCs) show motor dysfunctions and recover their function following reconstitution with A2A receptor and systemic administration with A2A agonists.

The use of PDRN played an effective anti-inflammatory response in various experimental models, demonstrating that adenosine receptors might be considered as important targets for anti-inflammatory action. Consistent with this point of view, PDRN has been effective following SCI, not only reducing pro-inflammatory cytokines but also ameliorating spinal cord structure. Twenty-four hours after SCI, clear signs of cell suffering were present, with fragmented and interrupted axonal and dendritic projection. PDRN preserved cell structure and reduced apoptotic rate, as demonstrated by the decreased expression of the pro-apoptotic protein, in favor of the anti-apoptotic one. In particular, PDRN administration reduced BAX expression and caused a significant increase of Bcl-2 expression compared to SCI animals, thus demonstrating that PDRN may affect the apoptotic process. In light of these results, however, we might also hypothesize that PDRN not only preserves cell death but also stimulates neurogenesis. Recently, neurogenesis and regeneration in the nervous system have been linked to the Wnt signaling, usually involved in developmental processes (David et al., 2010; Lambert et al., 2016). Wnt can be triggered by adenosine receptor activation, in fact the A2A is a G-protein coupled receptor that increases cAMP which is known to upregulate Wnt signaling (Fathi et al., 2017). The canonical Wnt pathway is activated when Wnt binds one member of the Frizzled (Fz) receptors (Wang et al., 2006); the interaction between Wnt and Fz requires the co-receptor low-density lipid receptor-related protein 6 (LRP6) and the recruitment of the scaffold protein Disheveled (Dvl). Once activated, the canonical Wnt pathway activates the intracellular β-catenin protein in the cytoplasm, which translocates into the nucleus acting as a transcriptional coactivator of transcription factors belonging to the TCF (T-cell specific transcription factor)/LEF (lymphoid enhancer-binding factor) family (Clevers and Nusse, 2012). When Wnt signaling is not activated, β-catenin remains accumulated in the cytoplasm forming a complex. Dickkopf1 (DKK1) is one of the main Wnt/β-catenin signaling inhibitors and, inhibiting Wnt activation, decreases β-catenin expression (MacDonald et al., 2009). Scientific evidences described Wnt signaling role in spinal cord and in synapses, showing that Wnt pathway may regulate neurogenesis and regeneration processes (David et al., 2010; Varela-Nallar et al., 2010).

Some reports have described an improved recovery of the CNS after injury through the activation of the Wnt/β-catenin signaling suggesting that the modulation of Wnt signaling may be a promising therapeutic strategy (Gao et al., 2016; Shen et al., 2017). Therefore, PDRN could be exploited for its dual effect obtained through the adenosine receptor: (i) the anti-inflammatory and (ii) the pro-regenerative one. In particular, within the Wnt family, Wnt-3a controls spinal cord dorsal interneuron specification and regulates neurogenesis (Murashov et al., 2005). Moreover, Wnt-3a displays mitogenic activities, promoting spinal cord growth (Megason and McMahon, 2002). Some studies showed the possible crosstalk between adenosine receptor activation and Wnt signaling, in fact, A2A receptor blockade reduced Wnt signaling in a murine model of bleomycin-induced fibrosis (Zhang et al., 2017), whereas A2A receptor activation, by using an agonist, stimulated Wnt pathway (Pizzino et al., 2017). Moreover, Hino et al. (2005) demonstrated that increased cAMP levels are released by A2A receptor stimulation and Wnt/β-catenin activation. Another study showed that A2AR stimulation with CGS21680, an A2AR agonist, promoted β-catenin activation and this effect was blocked by SCH58261, an A2AR antagonist (Shaikh et al., 2016).

So, the question was whether PDRN might also activate Wnt/β-catenin following adenosine receptor activation, treating experimental SCI. PDRN increased both Wnt3a and β-catenin mRNA expression, whereas DKK-1 mRNA expression, as expected, was reduced in PDRN treated group 24 h following SCI. These results revealed that also Wnt3a signaling is activated by adenosine receptor stimulation, confirming the hypothesis that PDRN may preserve neurons following SCI, as demonstrated by immunofluorescence staining and suggesting that it may also stimulate neurogenesis. Indeed, this latter hypothesis needs to be confirmed by further studies with the use of specific markers of neuronal regeneration. Another limitation of this study is the lack of a naïve group administered with PDRN to study the effects of this adenosine agonist on Wnt expression in uninjured spinal cord.

Moreover, DMPX was used to demonstrate that PDRN effects were related to A2A receptor binding: in fact PDRN effects on locomotor recovery and on Wnt pathway were abrogated following DMPX administration. These data confirm that PDRN is acting mainly through the adenosine receptor instead of its alternative mode of action, the so-called “salvage pathway” (Squadrito et al., 2017).

## Conclusion

This study describes the protective effect of PDRN following SCI: PDRN treatment significantly reduced the SCI-induced spinal cord tissue alteration, also improving motor function. The results of the present study enhance our understanding of PDRN mode of action, confirming PDRN anti-inflammatory activity and demonstrating that a crosstalk between Wnt/β-catenin signaling is possible by activation of adenosine receptors. Moreover, these data let us hypothesize that PDRN might promote neural repair through axonal regeneration and/or neurogenesis. However, further studies are required to elucidate PDRN mechanism of action in neurological disorders.

## Author Contributions

NI and AB: study design. NI, FM, AB, and GP: study conduct. GB and GV: data collection. NI, AB, LM, and VA: data analysis. NI, AB, EM, PB, and GA: data interpretation. NI, AB, and FS: drafting manuscript. NI, FS, and DA: revising the manuscript content. NI, VA, FM, GV, GP, LM, GB, GA, EM, PB, FS, DA, and AB: approving final version of manuscript.

## Conflict of Interest Statement

The author Francesco Squadrito has received research support from Mastelli for work on polydeoxyribonucleotide. Authors Francesco Squadrito, Alessandra Bitto and Domenica Altavilla are co-inventors on a patent describing therapeutic polydeoxyribonucleotide activity in chronic intestinal disease. Author Letteria Minutoli is a co-inventor on a patent describing therapeutic polydeoxyribonucleotide activity in testicular injury by torsion. The remaining authors declare that the research was conducted in the absence of any commercial or financial relationships that could be construed as a potential conflict of interest.
