# Corrigendum: Activation of A2A receptor by PDRN reduces neuronal damage and stimulates WNT/β-catenin driven neurogenesis in spinal cord injury

**DOI:** 10.3389/fphar.2022.1073726

**Published:** 2022-11-03

**Authors:** Natasha Irrera, Vincenzo Arcoraci, Federica Mannino, Giovanna Vermiglio, Giovanni Pallio, Letteria Minutoli, Gianluca Bagnato, Giuseppe Pio Anastasi, Emanuela Mazzon, Placido Bramanti, Francesco Squadrito, Domenica Altavilla, Alessandra Bitto

**Affiliations:** ^1^ Department of Clinical and Experimental Medicine, AOU Policlinico G. Martino, University of Messina, Messina, Italy; ^2^ Department of Biomedical and Dental Sciences and Morphological and Functional Sciences, AOU Policlinico G. Martino, University of Messina, Messina, Italy; ^3^ NIHR Leeds Biomedical Research Centre, Leeds Teaching Hospitals NHS Trust and Leeds Institute of Rheumatic and Musculoskeletal Medicine, University of Leeds, Leeds, United Kingdom; ^4^ IRCCS Centro Neurolesi “Bonino-Pulejo”, Messina, Italy

**Keywords:** adenosine receptors, inflammation, neurogenesis, polydeoxyribonucleotide, spinal cord injury

In the published article, the **Conflict of interest** statement was incorrect. The correct **Conflict of interest** statement appears below:

Conflict of interest

“The author FS has received research support from Mastelli for work on polydeoxyribonucleotide. Authors FS, AB and DA are co-inventors on a patent describing therapeutic polydeoxyribonucleotide activity in chronic intestinal disease. Author LM, FS and AB are co-inventor on a patent describing therapeutic polydeoxyribonucleotide activity in testicular injury by torsion.

The remaining authors declare that the research was conducted in the absence of any commercial or financial relationships that could be construed as a potential conflict of interest”.

The authors apologize for this error and state that this does not change the scientific conclusion of the article in any way. The original article has been updated.

